# A Potential Interplay between HDLs and Adiponectin in Promoting Endothelial Dysfunction in Obesity

**DOI:** 10.3390/biomedicines10061344

**Published:** 2022-06-07

**Authors:** Monica Zocchi, Matteo Della Porta, Federico Lombardoni, Roberta Scrimieri, Gian Vincenzo Zuccotti, Jeanette A. Maier, Roberta Cazzola

**Affiliations:** 1 Department of Biomedical and Clinical Sciences, Università degli Studi di Milano, 20157 Milan, Italy; monica.zocchi@unimi.it (M.Z.); matteodellaporta92@gmail.com (M.D.P.); federico.lombardoni@unimi.it (F.L.); roberta.scrimieri@unimi.it (R.S.); gianvincenzo.zuccotti@unimi.it (G.V.Z.); jeanette.maier@unimi.it (J.A.M.); 2 Department of Pediatrics, Ospedale dei Bambini, 20154 Milan, Italy

**Keywords:** low-grade chronic inflammation, HDLs, endothelial function, adiponectin, obesity

## Abstract

Obesity is an epidemic public health problem that has progressively worsened in recent decades and is associated with low-grade chronic inflammation (LGCI) in metabolic tissues and an increased risk of several diseases. In particular, LGCI alters metabolism and increases cardiovascular risk by impairing endothelial function and altering the functions of adiponectin and high-density lipoproteins (HDLs). Adiponectin is an adipokine involved in regulating energy metabolism and body composition. Serum adiponectin levels are reduced in obese individuals and negatively correlate with chronic sub-clinical inflammatory markers. HDLs are a heterogeneous and complex class of lipoproteins that can be dysfunctional in obesity. Adiponectin and HDLs are strictly interdependent, and the maintenance of their interplay is essential for vascular function. Since such a complex network of interactions is still overlooked in clinical settings, this review aims to highlight the mechanisms involved in the impairment of the HDLs/adiponectin axis in obese patients to predict the risk of cardiovascular diseases and activate preventive countermeasures. Here, we provide a narrative review of the role of LGCI in altering HDLs, adiponectin and endothelial functions in obesity to encourage new studies about their synergic effects on cardiovascular health and disease.

## 1. Introduction

In recent decades, the prevalence of obesity has grown steadily in the world population affecting virtually all ages and socioeconomic groups [1]. As obesity is one of the main factors contributing to the global burden of chronic non-communicable diseases and associated disabilities, a parallel increase in concern about its significant health and economic consequences has arisen.

Obesity is defined as abnormal or excessive fat accumulation in adipose tissue (AT) that presents a risk to health in individuals with a body mass index (BMI) over 30 kg/m^2^ [2] and is associated with low-grade chronic inflammation (LGCI) in metabolic tissues. LGCI directly contributes to insulin resistance (IR), metabolic syndrome (MetS) and type 2 diabetes mellitus (T2DM), and leads to several diseases, such as cardiovascular diseases (CVDs), cancer, chronic kidney disease, non-alcoholic fatty liver disease (NAFLD), autoimmune and neurodegenerative disorders [3]. LGCI alters metabolism and increases cardiovascular risk also by impairing endothelial function. In addition to traditional cardiovascular factors and genetic predisposition, in obese individuals, other parameters should be taken into account. In particular, HDLs can be dysfunctional, which means they loose their atheroprotective properties [4]. Moreover, the levels of adiponectin, an adipokine with anti-inflammatory and anti-atherogenic function, are decreased in obesity [5]. Some evidence exists about a possible interplay between HDLs and adiponectin, but, in spite of its relevance in clinical setting, this topic has been overlooked and more studies are needed. Because altered HDLs, adiponectin and LGCI cooperate in promoting endothelial dysfunction, which is the first step in atherogenesis, we here provide a narrative review about the potential interplay of these factors in triggering a pro-atherogenic endothelial phenotype in obesity.

## 2. Endothelial Function

The endothelium consists of 10^13^ cells which line all the vasculature, covering a surface of approximately 4000–7000 m^2^ [6]. It is a highly dynamic and active tissue, which provides a selective barrier to blood and maintains tissue homeostasis controlling the movements of macromolecules, including lipoproteins, to and from the tissues. Endothelial cells (ECs) also influence lipoprotein metabolism and function since they express a variety of lipoprotein receptors and several lipases that are bound to the extracellular matrix (lipoprotein lipase, LPL, hepatic lipase, HL and endothelial lipase, EL) and hydrolyze lipoprotein triglycerides (TGs) [7,8]. In addition, ECs are central in regulating vascular tone, platelet activation, coagulation, leukocyte trafficking, vascular smooth muscle cells proliferation and inflammation. In particular, ECs are a source and a target of inflammatory cytokines. It is also emerging that the endothelium plays a role in regulating metabolism. Indeed, nitric oxide (NO), constitutively released by ECs, not only regulates vascular tone, but also promotes insulin-dependent glucose utilization in the liver, skeletal muscle and AT [9]. Accordingly, asymmetric dimethylarginine, an endogenous NO synthase inhibitor, is increased in insulin-resistant or T2DM individuals [10,11].

Within the AT, a reciprocal communication exists between ECs and adipocytes. ECs transfer plasma constituents and biological signals via microvesicles to the adipocytes, which, conversely, release a plethora of bioactive molecules that model endothelial function [12]. It is also reported that ECs in the AT undergo an endothelial-mesenchymal transition, thus reducing vascularity and implementing fibrosis [9]. Of note, all the vessels are surrounded by AT, named perivascular adipose tissue (PVAT). PVAT is an established regulator of vascular function because it releases gas, such as NO and hydrogen sulfide, and adipokines, among which includes adiponectin, which influences endothelial function and vascular reactivity [13]. While in physiological conditions PVAT is vasculoprotective, its dysregulated structure and activity in obese subjects contribute to vascular dysfunction since it releases inflammatory mediators, among which tumor necrosis factor (TNF)-α [14] that readily promotes oxidative stress and the acquisition of an inflammatory phenotype in the endothelium. These features are typically associated with endothelial dysfunction (ED), the common denominator in numerous communicable and non-communicable diseases. ED is clinically assessed as a reduced endothelium-dependent relaxation, caused by a decreased NO availability [15]. In obese subjects, AT secretes lower amounts of adiponectin than in healthy individuals, and hypoadiponectinemia has been closely linked to impairment in endothelium-dependent vasodilation in both healthy subjects and patients with hypertension and T2DM [16,17,18]. A last issue to highlight is that obesity associated LGCI alters metabolism, including lipoprotein metabolism. In particular, HDLs, known to be vasculoprotective, are dysfunctional in obesity [19], with consequent reduction of NO, increase of oxidative species and inflammatory cytokines expression in ECs [19,20].

## 3. Adiponectin

Adiponectin is the most abundant endocrine peptide secreted by adipocytes and has widespread physiological activities derived from the combination of the endocrine actions of adipocyte-derived adiponectin with the autocrine or paracrine effects of local adiponectin produced by other cell types, such as skeletal and cardiac myocytes, osteoblasts and ECs. Unlike the majority of adipokines, serum adiponectin levels are reduced in obese individuals [5] and negatively correlate with chronic subclinical inflammation markers [21].

Adiponectin is involved in the regulation of energy metabolism and body composition, and serum adiponectin levels are inversely related not only to visceral fat accumulation, but also to the grade of IR and serum levels of glucose, insulin and TGs [22]. Adiponectin also exerts anti-inflammatory effects in ECs [5], promotes vascular homeostasis by increasing the levels of NO [23] and is involved in the crosstalk between AT, the immune system and the vascular wall [24].

Adiponectin secretion is mediated by proliferator-activated receptor gamma (PPARγ), a central regulator of adipocyte biology [25]. PPARγ is involved in the regulation of lipid metabolism and glucose homeostasis, and plays an important role in the cardiovascular system and in vascular development and homeostasis [26]. Moreover, PPARγ exerts an anti-inflammatory action and, accordingly, the transcription of the adiponectin gene in adipocytes is suppressed by pro-inflammatory cytokines, among which is TNF-α [27].

Adiponectin is found in serum as complexes of different molecular weight: low-molecular weight (LMW) trimer, medium molecular weight (MMW) hexamer and high-molecular weight (HMW) multimers that do not interconvert in vivo [5]. While trimeric and hexameric forms mostly regulate food intake [28], HMW forms of adiponectin mostly regulate insulin sensitivity, hepatic gluconeogenesis and other metabolic functions [29].

The effects of adiponectin are mediated by G-coupled receptors, which occur as two isoforms (AdipoR1 and AdipoR2). These receptors are ubiquitously expressed, AdipoR1 being particularly abundant in skeletal muscle and AdipoR2 in the liver [30]. Both adiponectin receptors are able to bind the multimerized fragments of adiponectin [31]. AdipoR1 has a high affinity for the trimer/hexamer forms, while the AdipoR2 preferentially binds to multimers [32]. Moreover, the adiponectin hexamer and multimer forms bind T-Cadherin, a membrane glycoprotein that can sequester adiponectin on the cell surface and is expressed mainly by endothelial and smooth muscle cells [33,34,35]. Of interest, it is after the binding of adiponectin to T-cadherin that ECs are stimulated to release microvesicles [36], thus emphasizing the complexity of the crosstalk between ECs and adipocytes [37].

AdipoR1 and AdipoR2 have also intrinsic ceramidase activity that is greatly amplified by the binding of adiponectin to its receptors and may be the underlying mechanism explaining many of the adiponectin-related phenotypes [38]. This enzyme cleaves fatty acids from ceramides, producing sphingosine, which, in turn, is phosphorylated by a sphingosine kinase to form sphingosine-1-phosphate (S1P). S1P is a pleiotropic lipid mediator that regulates several cell functions via high-affinity G protein-coupled receptors [38,39,40]. Adiponectin lowers hepatic ceramide content through enhanced ceramide catabolism with consequent production of S1P. Accordingly, in obesity, ceramide accumulates in various tissues, partly because of lower amounts of adiponectin [41].

**Figure 1:**
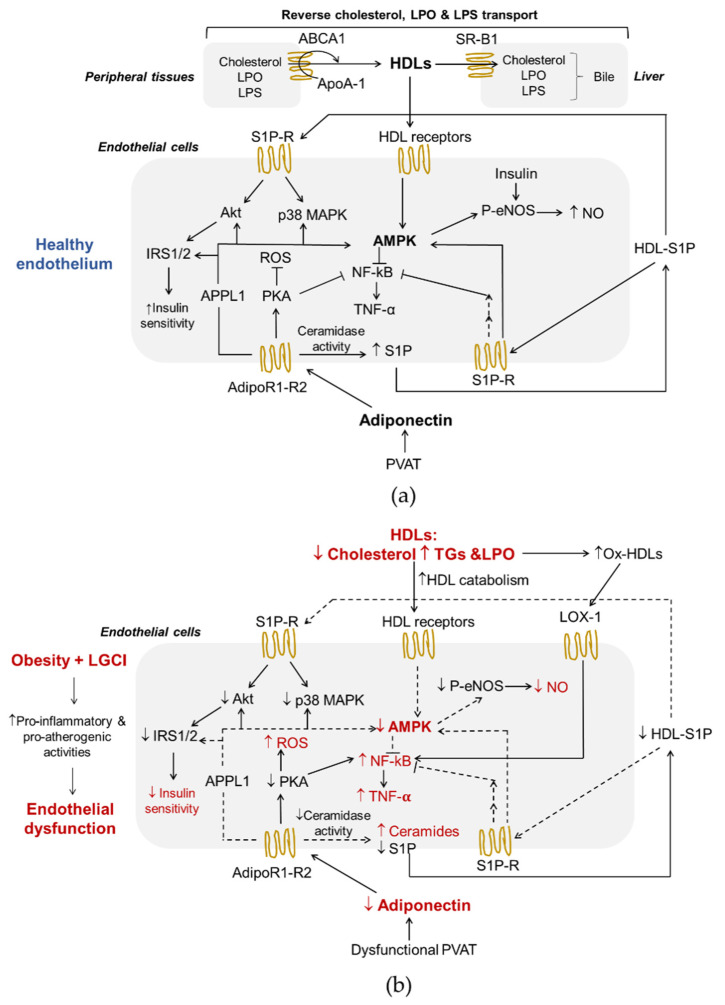
Adiponectin and HDLs interplay in endothelial function (**a**) and obesity-induced endothelial dysfunction (**b**). Both HDLs and adiponectin contribute to healthy endothelium in physiological conditions. In a background of obesity and low-grade inflammation, dysfunctional HDLs and the lower production of adiponectin by PVAT exert pro-inflammatory and pro-atherogenic effects on endothelial cells. Adiponectin receptor R1-R2, AdipoR1-R2; AMP-activated protein kinase, AMPK; apolipoprotein A-1, ApoA-1; ATP-binding cassette transporters A1, ABCA1; class B type 1 scavenger receptor, SR-B1; high-density lipoproteins, HDLs; insulin receptor substrate proteins 1/2, IRS1/2; lipopolysaccharide, LPS; lipoperoxides. LPO; oxidized receptor of low-density lipoproteins-1, LOX-1; low-grade chronic inflammation, LGCI; nitric oxide, NO; nuclear factor kappa-light-chain-enhancer of activated B cells, NF-kB; oxidized high density lipoproteins, ox-HDLs; perivascular adipose tissue, PVAT; phospho-endothelial nitric-oxide synthase, p-eNOS; phosphotyrosine interacting with PH domain and leucine zipper, APPL1; protein kinase A, PKA; protein kinase B, Akt; p38 mitogen activated protein kinase, p38 MAPK; reactive oxygen species, ROS; sphingosine-1-phosphate, S1P; sphingosine-1-phosphate receptor, S1P-R; triglycerides, TGs; tumor necrosis factor α, TNF-α;.

Adiponectin activates multiple signaling pathways, which mediate its metabolic actions and immunomodulatory effects. The binding of adiponectin to AdipoR1 and R2, through the recruitment of the adaptor protein phosphotyrosine interacting with PH domain and leucine zipper 1 (APPL1), triggers a series of tissue-dependent signal transduction events, including AMP-activated protein kinase (AMPK), p38 mitogen activated protein kinases (p38 MAPK), protein kinas A (PKA), peroxisome proliferator-activated receptor-α (PPARα), phosphatidylinositol-4,5-biophosphate 3-kinase (PI3K), insulin receptor substrate proteins 1 and 2 (IRS1/2)/protein kinase B (Akt) [5,42,43,44,45] (Figure 1a).

Adiponectin plays a metabolic role in maintaining energy homeostasis acting through phosphorylation and activation of AMPK [46,47]. AMPK is a metabolic sensor that is activated when ATP levels in the cells decrease. Its signaling regulates energy metabolism homeostasis [48] and can inhibit the inflammatory responses induced by the nuclear factor kappa-light-chain-enhancer of activated B cells (NF-κB) system [49]. In ECs, the AMPK pathway improves cell function through the activation of endothelial nitric oxide synthase (eNOS) and inhibits the secretion of inflammatory mediators (Figure 1a). In addition, the activation of protein kinase A (PKA) contributes to promote NO production and suppresses reactive oxygen species (ROS) generation and NF-κB signaling [50] (Figure 1a). In the liver, AMPK activation coordinates the partitioning of fatty acids between oxidative and biosynthetic pathways by increasing fatty acid oxidation capacity and inhibiting de novo lipogenesis [51], and hinders/blocks enzymes involved in gluconeogenesis promoting a reduction in blood glucose levels [52]. Moreover, the sequential activation of AMPK, p38 MAPK and PPARα increases the expression of enzymes involved in fatty acid oxidation [53]. p38 MAPK serves as a nexus for signal transduction and plays a vital role in numerous biological processes including the production of pro-inflammatory cytokines, such as IL-1β, TNF-α and IL-6 [54]. PPARα is a ligand-activated nuclear receptor highly expressed in the liver that acts as nutritional sensor and grants the adaptation of the rates of fatty acid catabolism, lipogenesis and ketone body synthesis, in response to feeding and starvation.

Insulin is a well-known regulator of glucose, protein and lipid metabolism. In addition, insulin promotes NO synthesis via eNOS [55] (Figure 1a). The binding of insulin to its receptors induces structural changes due to the auto-phosphorylation of tyrosine residues, followed by downstream events, such as the recruitment of different adaptor proteins (IRS1/2). Different types of insulin-dependent kinases, including Akt, AMPK and glycogen synthase kinase 3 (GSK-3), can phosphorylate and activate the IRS1/2 [56].

## 4. High-Density Lipoproteins

HDLs are a heterogeneous and complex class of lipoproteins with density ranging from 1.063–1.210 g/mL, considerable differences in size, shape, composition and function, produced mainly by the liver and, to a lesser extent, by the small intestine. In human plasma, the large, less dense (1.063–1.125 g/mL) lipid-enriched HDL2 and the small, dense (1.125–1.210 g/mL) protein-enriched HDL3 represent the two major sub-classes of HDLs [57]. HDLs contain several apolipoproteins (Apos) of which ApoA-I is quantitatively the most relevant and characterizes this lipoprotein class. Other Apos are ApoA-II, ApoA-IV, ApoC-I, ApoC-II, ApoC-III, ApoC-IV, ApoD, ApoE, ApoF, ApoH, ApoJ, ApoL-I and ApoM [57]. In addition, several enzymes circulate in the bloodstream associated with HDLs, including enzymes involved in lipoprotein remodeling (lecithin-cholesterol acyltransferase, LCAT, cholesterol ester transfer protein, cholesteryl ester transfer protein CETP, and phospholipid transfer protein, PLTP), paraoxonase-1 (PON-1) and lipopolysaccharide (LPS)-binding protein (LBP) [58]. The main lipids of HDLs are phospholipids (PLs) of which phosphatidylcholine (PC) and sphingomyelin (SM) are the main glycerophospholipids and sphingolipids, respectively. PLs modulate HDLs functions and are the precursors of a variety of regulatory molecules, including lysophospholipids and ceramides. In addition, S1P is transported in circulatory and interstitial fluids by HDLs-bound ApoM.

There are several interactions between HDLs and the endothelium (Figure 1a). First of all, reverse cholesterol transport (RCT), the ability to transport cholesterol from peripheral tissues back to the liver for excretion in the bile, is the best-known function of HDLs and a process that plays a central role in preventing endothelial dysfunction and atherosclerosis. Of interest, ECs express HDLs’ scavenger receptor B type I (SR-BI), the ATP-binding cassette transporters A1 and G1 (ABCA1 and ABCG1), and the ecto-F1-ATPase [59]. As shown in Figure 1a, upon the binding of HDLs to their receptors as well as to S1P receptors, various kinases, including Src, AMPK, p38 MAPK, PI3K and Akt, are activated [60,61]. As a result, HDLs enhance endothelial barrier-function and exert anti-inflammatory, anti-apoptotic and anti-adhesive properties [57,62,63,64]. In addition, HDLs reduce the cellular production of superoxide, an inactivator of the vasodilator NO, by decreasing the activity of endothelial nicotinamide adenine dinucleotide phosphate (NADPH) oxidase [65], thus preventing ED.

RCT begins with the formation of nascent HDLs particles, which consist mainly of ApoA-I (Figure 1a). Cholesterol efflux is mediated by the transporters ABCA1, ABCG1 and SR-B1. This step involves the interaction between lipid-free or lipid-free monomeric ApoA-I and ABCA1, while ABCG1 mediates the outflow of cellular cholesterol to lipidated HDL particles. The expression of the ABCA1 and ABCG1 genes is regulated at the transcriptional level by the liver X receptors (LXRs)-α and β [66]. Like ABCG1, SR-B1 in peripheral cells may also promote cholesterol outflow to mature HDLs particles, but its role in the RCT pathway is particularly important in the liver where it mediates the selective uptake of cholesteryl esters from HDLs [24]. HDLs also influence triglyceridemia because of their regulatory role on HL activity. HL binds to proteoglycans on the cell surface of hepatocytes and hepatic ECs. HDLs bind to HL and release the enzyme into the circulation where it hydrolyses TGs and PLs of plasma lipoproteins [67]. HDL2 is more effective in displacing proteoglycan-bound HL than the HDL3. In addition, both in vivo and in vitro models suggest that HDLs promote an increase in the production of adiponectin from AT in a P13K-dependent manner [68].

Another well-known function of HDLs is their role as anti-inflammatory regulators exerted through interactions with both the vascular endothelium and circulating inflammatory cells [69]. As mentioned above, HDLs reduce the expression of endothelial adhesion molecules in response to inflammatory mediators and the migration of monocytes into the vascular wall, simultaneously exploiting their antioxidant activities [57]. HDLs prevent the induction of endothelial 32-kDa putative cysteine protease (CPP32)-like protease, resulting in a decrease in the activity of TNF-α, and, consequently, reduce the apoptotic rate of these cells [70]. Moreover, HDLs participate in a mechanism of intercellular communication involving the transport and delivery of specific microRNAs (miRNAs), small non-coding RNAs that post-transcriptionally regulate gene expression through translational inhibition and mRNA destabilization [71]. It has been shown that the transfer of miRNA-223 from HDLs into ECs reduces inflammation by suppressing the expression of intercellular adhesion molecule 1 (ICAM-1) [72].

The anti-inflammatory properties of HDLs may be due also to their ability to neutralize bacterial products, such as LPS (Figure 1a). LPS is a bacterial endotoxin with powerful pro-inflammatory activity that can reach the systemic circulation even during the absorption of nutrients in much smaller quantities than those associated with a bacterial infection, but sufficient to contribute to LGCI [73]. Moreover, LPS decreases HDL cholesterol (HDL-C) and adiponectin levels in vivo [68] and directly and indirectly participates in the inflammatory reaction in AT during obesity [74]. The exposure of ECs to this endotoxin results in endothelial activation and production of various pro-inflammatory mediators, and, ultimately, in cellular injury [75]. Interestingly, very recently, Han et al. have demonstrated that intestine-derived HDL3 traverses the portal vein complexed with LPS-binding protein preventing LPS activation of liver macrophages and supporting extracellular inactivation of this endotoxin [58].

Finally, the antioxidant activities of HDLs prevent ED via endothelial ABCG1-mediated efflux of cholesterol and 7-oxysterols [76] and the inhibition of lipid peroxide accumulation because of PON-1 activity. HDLs are the major carrier in the circulation of PON-1, an esterase characterized by three enzymatic activities (lactonase, arylesterase and paraoxonase) that is involved in drug metabolism, and possesses antioxidant and anti-inflammatory properties [77]. The esterase activities of PON-1 allow the removal of peroxidized fatty acids from PLs, limiting damage resulting from oxidative stress. PON-1 hydrolyses also lactones, including homocysteine thiolactone, a toxic metabolite of homocysteine, which, by modifying protein lysine residues, leads to cell death, altered vessel structure, chronic inflammation, autoimmune response and atherosclerosis [78]. This PON-1 activity is probably involved in the mechanisms by which HDLs activate eNOS in an inflammatory environment [60]. Another important antioxidant activity of HDLs can be ascribed to their reverse transport of lipid peroxides [79] (Figure 1a). In fact, HDLs can acquire lipid peroxides from low-density lipoproteins (LDLs) and cell membranes holding them in an environment where they may be safely hydrolyzed and from which they may be released to the liver for elimination.

## 5. Obesity, Inflammation and Lipid Metabolism Dysfunction

**Figure 2:**
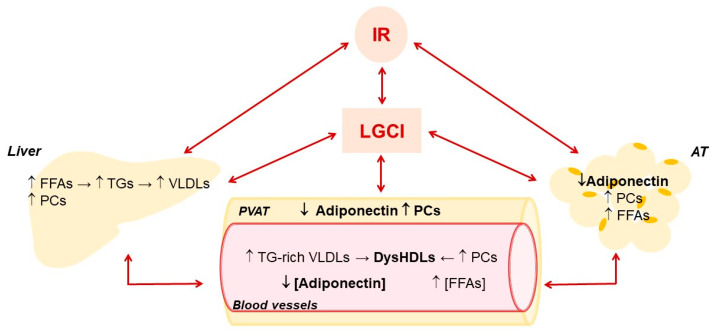
Main effects of low-grade chronic inflammation and insulin resistance on liver and adipose tissue metabolism and their influence on HDLs and adiponectin in blood vessels. Adipose tissue, AT; Free fatty acids, FFAs; insulin resistance, IR; low-grade chronic inflammation, LGCI; proinflammatory cytokines, PCs; perivascular adipose tissue, PVAT; triglycerides, TGs; very low-density lipoproteins, VLDLs.

Frequent nutrient overload not only leads to being overweight and obese, but also changes metabolism. It determines an increase of lipid accumulation and glycolytic ATP synthesis associated with decreased mitochondrial biogenesis and excessive ROS production. In addition, there is an activation of inflammatory responses in AT, liver, skeletal muscle, pancreas and hypothalamus that contribute to decreased insulin sensitivity and systemic IR in obese individuals (Figure 2), although not all obese subjects become insulin resistant [80,81,82]. In obesity, IR is associated with hyperinsulinemia and hyperglycemia, increased visceral adiposity, metabolic dyslipidemia with high triglyceridemia and low HDL-C levels, and hypertension, characteristics collectively referred to as the MetS [83].

In obese liver, hepatocytes and endothelial sinusoidal cells accumulate ROS and release pro-inflammatory cytokines, and Kupffer and stellate cells are activated and acquire a pro-inflammatory phenotype [84]. The expansion of fat mass in obesity is accompanied by the reduction of adiponectin production by adipocytes and the infiltration into AT of immune cells of which macrophages are the most abundant. The AT macrophages (ATMs) are classified into M1 (pro-inflammatory macrophages) and M2 phenotype (anti-inflammatory macrophage). In a physiological state, a balance exists between M1 and M2 phenotypes, while in obesity there is polarization of ATM toward their M1 phenotype. Therefore, pro-inflammatory ATMs release chemokine, such as monocyte chemoattractant protein-1 (MCP-1), and pro-inflammatory cytokines, such as IL-1β, IL-6 and TNF-α, all contributing to LGCI [85]. These cytokines cause the recruitment of more macrophages and circulate in the blood to propagate inflammation to the other tissues [86]. Consequently, obesity is characterized by an increased concentration of circulating pro-inflammatory cytokines responsible for LGCI, which is recognized as a major cause of decreased insulin sensitivity and IR, regardless of total body fat mass [80,81,82]. Adiponectin suppresses M1 inflammatory activation and promotes M2 macrophage polarization. Therefore, reduced adiponectin in obesity contributes to LGCI [87,88]. Even if the exact etiology of AT inflammation has not been fully clarified yet, inflammation arising from the AT is the common factor that links obesity, diet, physical inactivity and metabolic diseases, mainly through the promotion of peripheral IR and the alteration of circulating adipokines and lipid profile [80,81,82,89,90,91,92].

Among pro-inflammatory cytokines, TNF-α plays a key role. In adipocytes, TNF-α suppresses the transcription of the adiponectin gene [27] and impairs insulin receptor phosphorylation and kinase pathways by activating NF-κB and c-Jun NH2-terminal kinase (JNK) signaling, thereby promoting IR [85]. In the hepatocytes, TNF-α increases fatty acid synthesis, by raising the level of citrate and cholesterol synthesis through the induction of 3-hydroxy-3-methylglutaryl CoA reductase activity, and enhances LPL mRNA levels in association with an excessive LPL activity in plasma [93]. TNFα also impairs endothelial response to insulin, by interfering with IRS-1 and AMPK signaling, thus blocking NO production [94,95]. Activation of AMPK inhibits TNFα’s ability to cause inflammation and IR in various cell types, while decreased AMPK activity improves its ability to do so. On the other hand, insulin and its receptor seem able to decrease circulating adiponectin levels because subjects with severe IR due to insulin receptor genetic abnormalities or subjects with anti-insulin receptor autoantibodies are characterized by increased circulating adiponectin levels [96], and insulin administration reduces adiponectin levels in healthy individuals [97]. This last observation suggests a vicious circle during the early stages of hyperinsulinemia, whereby high insulin levels lead to a reduction in adiponectin levels, which in turn further decreases insulin sensitivity and increases circulating insulin levels to maintain glucose homeostasis. On the other hand, as IR progresses to T2DM, adiponectin resistance and subsequent compensatory hyperadiponectinemia might occur [98]. This situation is often referred to as “the adiponectin paradox” [35].

The alteration of hepatic metabolism promoted by LGCI is also negatively conditioned by the high portal influx of fatty acids endorsed by IR in AT, which leads to a further increase in the synthesis of TGs and Apo-B and, consequently, of very low-density lipoproteins (VLDLs) (Figure 2). The resulting hypertriglyceridemia increases the exchange between the TGs of VLDLs and cholesterol esters of LDLs and HDLs operated by CETP, producing more atherogenic TGs-rich LDLs and HDLs. TGs-enrichment of HDLs inhibits their ability to move HL from the cell surface of hepatocytes and hepatic ECs [99], contributing to hypertriglyceridemia. In addition, compared to normal HDLs, TGs-enriched HDLs are smaller and with higher catabolic rates, thereby lowering HDL-C levels. [100]. Last but not least, these HDLs are more susceptible to lipid peroxidation [101] and consequently in a pro-oxidant environment, such as the one promoted by LGCI, they easily turn into ox-HDLs. The alteration of lipid and lipoprotein metabolism promoted by IR increases the risk of NAFLD, MetS, T2DM and CVDs [102].

Finally, by increasing the plasma levels of IL-6, LGCI promotes hepatic production of serum amyloid A, which replaces ApoA-I and PON-1 in HDLs [57]. Moreover, in obese subject, activated neutrophils within the atherosclerotic plaque release myeloperoxidase (MPO), catalyzing the formation of ROS, i.e., hypochlorite, that can oxidize HDLs [103]. MPO associates with circulating HDLs through the formation of a ternary complex with ApoA-I and PON-1 [104]. PON-1 partially inhibits MPO activity, while MPO inactivates PON-1 [105]. In accordance with these observations, in obese subjects, a reduction in PON-1 activity has been observed, which probably results from a reduction in both the concentration of its plasma carrier (HDLs) and its enzymatic activities [106]. The final result is the loss of HDLs ability to inhibit lipoprotein oxidation and increased oxidative stress [107].

## 6. Interplay between Adiponectin and HDLs in Endothelial Function and Obesity-Associated ED

Adiponectin and HDLs are strictly interdependent and the maintenance of their interplay is essential for a healthy endothelium (Figure 1a) [4]. Dysfunctional HDLs and lower levels of adiponectin observed in obesity and related to LGCI significantly contribute to endothelial dysfunction due to pro-inflammatory and pro-atherogenic effects (Figure 1b).

**Figure 3:**
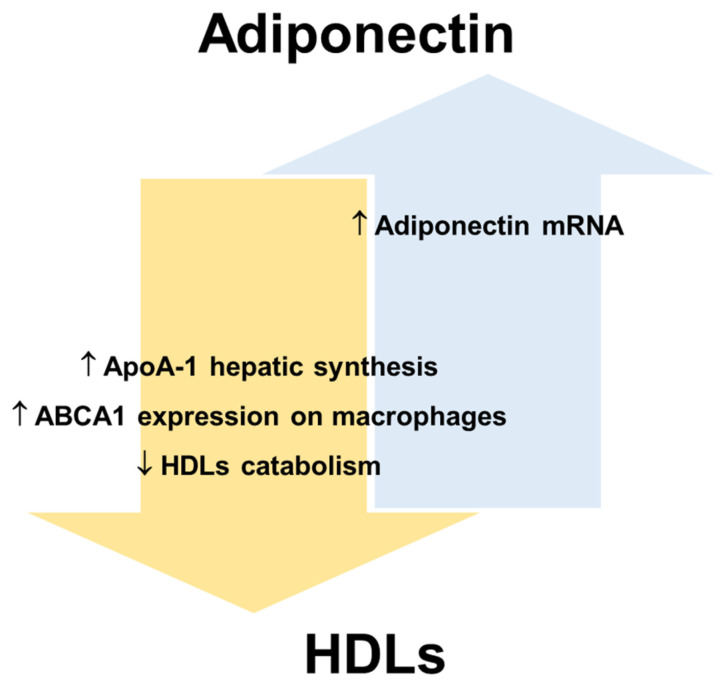
Reciprocal regulation of adiponectin and HDLs. Apolipoprotein A-1, ApoA-1; ATP-binding cassette transporters A1, ABCA1; high-density lipoproteins, HDLs.

HDLs and adiponectin reciprocally regulate their levels and metabolism (Figure 3). HDLs can enhance circulating adiponectin levels [68,108], while the amount of circulating adiponectin is an independent predictor of cellular cholesterol efflux capacity in humans [24]. Dias et al. [109] showed that elevated adiponectin levels are associated with a lower reduction in HDLs function assessed by measuring ApoA-I levels, particle size, cholesterol content and antioxidant capacity in T2DM patients. Nonetheless, the mechanisms that link the metabolism of adiponectin and lipoproteins have not been fully elucidated mainly because analytical difficulties (explained in Section 7) complicate data collection and interpretation.

In this regard, human and animal studies suggest that adiponectin promotes not only cellular cholesterol efflux but also HDLs biogenesis [24], while reducing the catabolism of HDLs/ApoA-I and the clearance rate of VLDLs [24,110]. Several human studies have shown that the levels of adiponectin and HDL-C are positively correlated in serum (revised in [108]). Marsche et al. observed in adult obese subjects a positive correlation between reduced plasma adiponectin and cholesterol efflux capacity of HDLs, independently of sex, BMI, fat distribution, blood pressure, and kidney and liver functions [111]. A recent study [112] shows that the levels of HDL-C significantly improve in obese individuals after bariatric surgery, which determines a rapid reduction of AT and consequently an increase of adiponectin production. In particular, the data show that bariatric surgery promotes an improvement of ApoA-I and adiponectin levels in parallel with an amelioration of cholesterol efflux capacity. For this reason, the increase in adiponectin production, as a crucial modulator of ApoA-I synthesis and hence of HDLs function, may represent a mechanism for decreasing cardiovascular risk associated with obesity [112]. HDL-C itself is considered a positive factor for endothelial health and is inversely related to CVD risk. Results from human studies suggest that low plasma adiponectin levels are associated with decreased LPL mass and activity [113,114]. LPL is a lipolytic enzyme that hydrolyses the TGs of VLDLs and chylomicrons and a decrease in its activity is associated with an increase in plasma TGs and a decrease in HDL-C levels [115]. Therefore, adiponectin could indirectly maintain HDLs number and function by preventing the enrichment of HDLs in TGs. Importantly, an increase in TGs-rich lipoproteins also leads to ED by dysregulating the cytokine network and decreasing insulin-induced NO synthesis [116].

Another possible interaction could concern the regulation of ceramide concentration in EC membranes and the production and transport of S1P. HDLs transport significant amounts of ceramides. However, how ceramides are incorporated into HDLs particles is still unknown. It has been hypothesized that PLTP and CETP might transfer ceramides from ApoB-lipoproteins (where ceramides are incorporated during VLDLs assembly) to HDLs and/or that HDLs might directly accept ceramides from plasma membranes [117]. On the other hand, the ceramidase activity of AdipoRs and the activation of neutral ceramidase by adiponectin decreases ceramide concentration in the membranes of ECs and produces S1P, which also mediates angiogenesis, induces the formation of tight junctions between ECs and plays an important role in maintaining the endothelial barrier. Moreover, S1P can be released into the blood, thus contributing to the pool of circulating S1P [118,119,120,121]. In addition, the binding of adiponectin to T-cadherin triggers the formation and release of ceramide-containing exosomes, thus lowering EC ceramide content [122]. Therefore, vascular endothelium is a significant source of circulating S1P because of adiponectin activity and HDLs are an important carrier of this bioactive lipid in serum. LGCI leads to increased production of ceramides by the activation of sphingomyelinase and the accumulation of ceramides in ECs causes ROS overproduction and ED [23].

In hyperglycemic and dyslipidemic subjects, significantly higher levels of oxidative stress and CVDs risk markers are observed than in healthy subjects, concomitantly with significantly lower levels of adiponectin [123].

Both chronic inflammation and oxidative stress promote peroxidative damages of proteins and lipids in HDLs resulting in the formation of oxidized HDLs (ox-HDLs) characterized by a loss antioxidant and anti-inflammatory activities, reduced capacity to remove cholesterol from macrophages and other pro-atherosclerotic effects with negative repercussions also on endothelial function [124,125,126,127]. The peroxidation of HDL-polyunsaturated fatty acids results in the formation of oxidized lipids and peroxidation end products, including various bioactive aldehydes, such as malondialdehyde (MDA), 4-hydroxynonenal (4-HNE) and acrolein [64]. These aldehydes are known to contribute to numerous pathologies through the alteration of proteomic, genomic, cellular signaling and metabolic processes [128] and to be able to modify apolipoproteins, making atherogenic the LDLs [129] and the HDLs less effective in counteracting atherogenesis [128]. Moreover, ox-HDLs, like ox-LDLs, induce an increase in the concentration of the oxidized receptor of low-density lipoproteins-1 (LOX-1) in the plasma membrane of ECs [20] (Figure 1b). LOX-1 activation by ox-LDLs/HDLs and other ligands causes ED by activating NF-κB and the subsequent induction of adhesion molecules and endothelial apoptosis [130], and triggering endothelial PKCβII activation, which in turn inhibits eNOS-activating pathways and, consequently, eNOS-dependent NO production [131] (Figure 1b). It has been shown that adiponectin selectively binds and inhibits the uptake of oxidized LDLs (oxLDLs) but not of native LDLs by LOX-1 [132]. Unfortunately, to the best of our knowledge, studies have not been published that demonstrate this effect also on ox-HDLs. However, subjects with morbid obesity show an increased level of circulating ox-HDLs, a markedly reduced amount of adiponectin and an increased level of circulating ECs, reliable indicators of vascular injury and damage [133]. Coherently, the number of endothelial progenitor cells colonies was reduced in these patients, suggesting the presence of an early endothelial stem cell dysfunction and a decreased endothelium repair capacity [134].

Finally, ox-HDLs significantly contribute to the trans-differentiation of vascular smooth muscle cells (VSMCs) into osteoblasts by enhancing the activity of alkaline phosphatase and calcium deposition, thus promoting vascular calcification and atherosclerosis plaques progression [135]. Interestingly, adiponectin contributes to anti-atherogenic phenotype also by counteracting vascular calcification. In particular, some data show that adiponectin reduces calcium deposition in human VSMCs by preventing ox-HDLs-related production of IL-6, WNT-5a and NF-ĸβ (p65) [136]. Moreover, adiponectin reduces TNF-α -induced calcification in VSMCs through the activation of AMPK [137] and prevents the osteogenic differentiation of VSMCs by downregulating the expression of the osteogenic transcription factor Osterix through the inhibition of STAT3 phosphorylation and nuclear transport [138].

## 7. Potential Biomarkers of Adiponectin and HDLs Functions

LGCI promotes lowering cholesterol concentration and impairing functions in HDLs and hypoadiponectinemia in obesity. Hypodiponectinemia, as reported above, is associated with LGCI in obesity and metabolic disorders such as T2DM and atherosclerosis. However, recent studies have shown that high serum levels of adiponectin are positively associated with the severity of inflammation and pathological progression in inflammatory diseases such as rheumatoid arthritis, chronic kidney disease and inflammatory bowel disease. On the other hand, low HDL-C levels predict increased CVD risk, particularly in healthy individuals with no history of cardiovascular events and, as with adiponectin [139], a U-shape relationship between HDL-C and CVDs has been shown [140]. Furthermore, drugs that increase HDL-C have failed to show improved cardiovascular outcomes [141].

Since the discovery of adiponectin and its isoforms, many different assays have been developed to define the serum level of adiponectin. Several old studies suggest that HMW and total adiponectin are biologically active independently of each other, with varying ratios between the two [97,142,143,144,145,146]. The ratio of HMW to total adiponectin was defined by Pajvani et al. as the adiponectin sensitivity index (ASI) on the basis of the correlation of this index with insulin sensitivity in a cohort of patients with T2DM [146]. Moreover, an association has been found between blood levels of HMW adiponectin and age, HDL-C and visceral adiposity [29] as well as with ED [147]. On the other hand, no difference between total adiponectin and HMW were described in another association studies performed in different clinical conditions [148,149]. In addition, more recently, van Alden et al. have shown that ASI correlates with the total concentration of adiponectin regardless of various clinical conditions [150], and therefore total and HMW adiponectin have similar utility in the assessment of adiponectin levels in blood. Unfortunately, to date, no gold standard for adiponectin measurement in blood is available and analytical issues could help explain the contradictory results reported in association studies. Therefore, the discussion of the biological relevance of adiponectin isoforms is still open.

Considering the several functions of HDLs and the numerous changes of their composition induced by LGCI, it is quite evident that the function of HDLs cannot be assessed by considering only their cholesterol levels. The discovery almost 30 years ago that HDLs can be converted from anti-inflammatory particles into proinflammatory particles during an acute response in humans [151] and a series of subsequent studies (revised in [152]) have brought out a new perspective on HDLs, namely that structural changes closely related to the functional state of HDLs and the assessment of HDLs function may be more relevant than cholesterol levels to represent HDLs effects on the organism [57,153,154,155,156,157]. This led initially to the study of key structural factors such as ApoA-I, MPO and PON-1 as potential markers of the functional capacity of HDLs [152,158,159,160], and subsequently to a more complete evaluation of the composition of these lipoproteins with omics techniques, such as lipidomic and proteomic [161,162]. Furthermore, in recent decades, various tests have been developed to measure the functionality of HDLs, such as measuring the capacity of cholesterol efflux, the antioxidant and anti-inflammatory properties and the ability to stimulate NO production and the proliferation of endothelial progenitor cells [152]. More recently, S1P has been investigated regarding the functional properties of this lipoprotein [163,164]. As above mentioned, vascular endothelium is a significant source of circulating S1P because of adiponectin activity and HDLs are an important carrier of this bioactive lipid in serum. However, data on the predictive value of circulating levels of total S1P or HDL-bound S1P as potential CVDs biomarkers are scarce [165].

Unfortunately, these techniques are limited to experimental studies and decades of research on new HDLs biomarkers have not led to univocal and reliable results.

## 8. Conclusions

Due to its significant comorbidities, mortality and costs, obesity must be treated and, above all, prevented (especially in children). The treatment of obesity starts with calorie restriction obtained with different dietary patterns and can go as far as bariatric surgery. In obesity, LGCI and oxidative stress could develop, which contribute to the promotion of associated comorbidities. The availability of biomarkers indicative of metabolic dysfunctions promoted by LGCI and oxidative stress would certainly be very useful to evaluate the effectiveness of therapies and non-pharmacological measures in reducing the risk of developing pathologies. Adiponectin and HDLs can reciprocally regulate their serum levels and activities, and both can modulate LGCI and oxidative stress and contribute to the maintenance of endothelial functions. Considering the several functions of HDLs and the numerous changes of their composition induced by LGCI, oxidative stress and many pathological states, it is quite evident that measuring cholesterol levels does not suffice. Therefore, it is not surprising that HDL-C levels show a relationship with adverse events and longevity described by a U-shaped curve. However, biomarkers for monitoring dysfunctional HDLs in clinical practice have not yet been established. Adiponectin activates multiple signaling pathways, which mediate its metabolic actions and immunomodulatory effects. As with HDLs, serum adiponectin levels show a relationship with adverse events and longevity described by a U-shaped curve. In the context of obesity, a panel of biomarkers that includes parameters indicative of the function of HDLs, adiponectin and endothelium can certainly help to better characterize their specific functions and their synergistic effects in cardiovascular health and/or disease. More research should be fostered to develop novel diagnostic tools and investigate the complex world of adiponectin and HDLs in obesity.

## Data Availability

Data sharing is not applicable to this article.
